# Safety and efficacy of HSP90 inhibitor ganetespib for neoadjuvant treatment of stage II/III breast cancer

**DOI:** 10.1038/s41523-022-00493-z

**Published:** 2022-12-01

**Authors:** Julie E. Lang, Andres Forero-Torres, Douglas Yee, Christina Yau, Denise Wolf, John Park, Barbara A. Parker, A. Jo Chien, Anne M. Wallace, Rashmi Murthy, Kathy S. Albain, Erin D. Ellis, Heather Beckwith, Barbara B. Haley, Anthony D. Elias, Judy C. Boughey, Rachel L. Yung, Claudine Isaacs, Amy S. Clark, Hyo S. Han, Rita Nanda, Qamar J. Khan, Kristen K. Edmiston, Erica Stringer-Reasor, Elissa Price, Bonnie Joe, Minetta C. Liu, Lamorna Brown-Swigart, Emanuel F. Petricoin, Julia D. Wulfkuhle, Meredith Buxton, Julia L. Clennell, Ashish Sanil, Scott Berry, Smita M. Asare, Amy Wilson, Gillian L. Hirst, Ruby Singhrao, Adam L. Asare, Jeffrey B. Matthews, Michelle Melisko, Jane Perlmutter, Hope S. Rugo, W. Fraser Symmans, Laura J. van ‘t Veer, Nola M. Hylton, Angela M. DeMichele, Donald A. Berry, Laura J. Esserman

**Affiliations:** 1 University of Southern California, Los Angeles, USA; 2 University of Alabama at Birmingham, Birmingham, USA; 3 University of Minnesota, Minneapolis, USA; 4 University of California San Francisco, San Francisco, USA; 5 University of California San Diego, San Diego, USA; 6 University of Texas MD Anderson Cancer Center, Houston, USA; 7 Loyola University Chicago Stritch School of Medicine, Maywood, USA; 8 Swedish Cancer Institute, Seattle, USA; 9 University of Texas Southwestern, Dallas, USA; 10 University of Colorado, Boulder, USA; 11 Mayo Clinic Rochester, Rochester, USA; 12 University of Washington, Seattle, USA; 13 University of Georgetown, Washington, DC, USA; 14 University of Pennsylvania, Philadelphia, USA; 15 Moffitt Cancer Center, Tampa, USA; 16 University of Chicago, Chicago, USA; 17 University of Kansas, Lawrence, USA; 18 Inova Health System, Virginia, USA; 19 George Mason University, Fairfax, USA; 20 Berry Consultants, LLC, Austin, USA; 21 Quantum Leap Healthcare Collaborative, San Francisco, USA; 22 Gemini Group, Michigan, USA

**Keywords:** Targeted therapies, Breast cancer, Prognostic markers

## Abstract

HSP90 inhibitors destabilize oncoproteins associated with cell cycle, angiogenesis, RAS-MAPK activity, histone modification, kinases and growth factors. We evaluated the HSP90-inhibitor ganetespib in combination with standard chemotherapy in patients with high-risk early-stage breast cancer. I-SPY2 is a multicenter, phase II adaptively randomized neoadjuvant (NAC) clinical trial enrolling patients with stage II-III breast cancer with tumors 2.5 cm or larger on the basis of hormone receptors (HR), HER2 and Mammaprint status. Multiple novel investigational agents plus standard chemotherapy are evaluated in parallel for the primary endpoint of pathologic complete response (pCR). Patients with HER2-negative breast cancer were eligible for randomization to ganetespib from October 2014 to October 2015. Of 233 women included in the final analysis, 140 were randomized to the standard NAC control; 93 were randomized to receive 150 mg/m^2^ ganetespib every 3 weeks with weekly paclitaxel over 12 weeks, followed by AC. Arms were balanced for hormone receptor status (51–52% HR-positive). Ganetespib did not graduate in any of the biomarker signatures studied before reaching maximum enrollment. Final estimated pCR rates were 26% vs. 18% HER2-negative, 38% vs. 22% HR-negative/HER2-negative, and 15% vs. 14% HR-positive/HER2-negative for ganetespib vs control, respectively. The predicted probability of success in phase 3 testing was 47% HER2-negative, 72% HR-negative/HER2-negative, and 19% HR-positive/HER2-negative. Ganetespib added to standard therapy is unlikely to yield substantially higher pCR rates in HER2-negative breast cancer compared to standard NAC, and neither HSP90 pathway nor replicative stress expression markers predicted response. HSP90 inhibitors remain of limited clinical interest in breast cancer, potentially in other clinical settings such as HER2-positive disease or in combination with anti-PD1 neoadjuvant chemotherapy in triple negative breast cancer.

Trial registration: www.clinicaltrials.gov/ct2/show/NCT01042379

## Introduction

The Heat Shock Protein 90 (HSP90) protein functions as an adenosine triphosphate-dependent molecular chaperone, helping promote maturation and stability of multiple cellular proteins known as “clients.” Many of these clients are oncoproteins associated with cancer cell proliferation and immortalization, regulation of cell cycle progression, neovascularization, and apoptosis of cancer cells^1,2^. Among these client proteins are steroid hormone receptors for estrogen and progesterone, receptor tyrosine kinases (e.g., HER2), epidermal growth factor receptor (EGFR) and intermediates of oncogenic signaling cascades (AKT and RAF1) relevant to various breast cancer subtypes^3^. Based on extensive preclinical evaluation, HSP90 has been considered as a therapeutic target for many cancers, including those of the breast^4^.

Ganetespib is a second-generation small molecule inhibitor of HSP90 with potent inhibitory effects on HSP90-dependent oncoproteins relevant to breast cancer pathogenesis. It has shown potent antitumor activity in both in vitro and in vivo models^5–9^. In initial phase 1 and 2 clinical trials in solid tumors, ganetespib was well tolerated^10–12^. It has been evaluated in patients with metastatic breast cancer, where it failed to meet endpoints, although some activity was observed in trastuzumab-refractory HER2-positive and in triple negative breast cancer (TNBC)^13^. Ganetespib also appears to be well tolerated when combined with docetaxel chemotherapy, with grade 3/4 adverse events similar to docetaxel alone^14^.

Thus we evaluated ganetespib in combination with paclitaxel followed by doxorubicin/cyclophosphamide as neoadjuvant treatment for early-stage breast cancer in the I-SPY2 Trial. The ganetespib arm was open only to HER2-negative patients, as I-SPY2 was focused on evaluating specific HER2-targeted regimens for HER2-positive patients during this time period. I-SPY2 is a biomarker rich trial and as such, we evaluated 18 expression-based biomarkers in HSP90, GR/efflux, replicative stress, and immune pathways previously shown to associate with response to HSP90-inhibiton^15–20^ in an effort to identify predictive markers of response to ganetespib treatment.

## Results

### Patients and disease characteristics

**Fig. 1:**
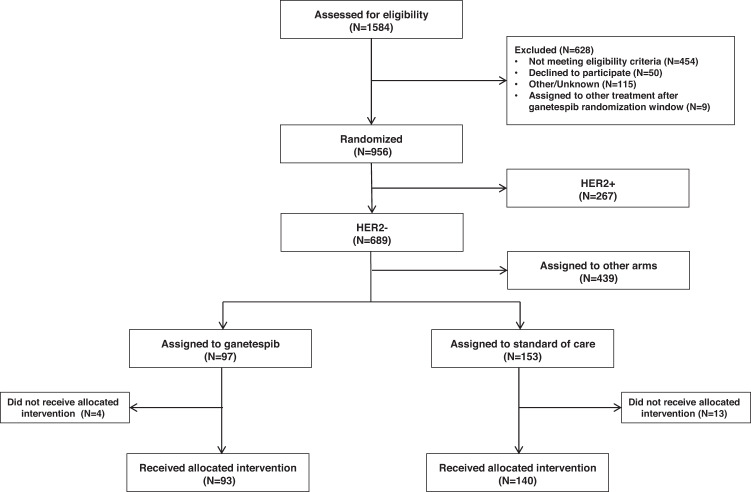
CONSORT diagram. Details of the screening, randomization, and treatment for patients assigned to ganetespib or standard of care are provided.

Ganetespib was open for enrollment from October 13, 2014 to October 3, 2015. From the start of the trial in March 2010 through October 3, 956 patients were eligible to be randomized in 6 different research arms, including ganetespib, ganitumab, pertuzumab, T-DM1 and pertuzumab, PLX3397 and control (Fig. 1). Of these, 97 HER2-negative were assigned to the ganetespib arm and 153 were randomized to the control arm. As shown in Fig. 1, four patients in the ganetespib arm and 13 patients in the control arm did not receive the assigned intervention and are not included in the analysis, yielding a final study population of 93 evaluable patients in the ganetespib arm and 140 in the control arm who serve as contemporary controls. Overall baseline patient characteristics were mostly similar between the experimental and control arms (Table 1). However, more patients enrolled in the ganetespib arm were Mammaprint^21^ High2 (MP2, 66% vs. 46%, chi-square test *p* = 0.002), despite having equivalent rates of HR-positive disease compared to control. Patients randomized to ganetespib had lower rates of baseline palpable axillary nodes (28% vs. 46%, chi-square test *p* = 0.017,). There were 10 patients (4 in ganetespib and 6 in control arm) whose pCR results were unavailable; these patients did not have surgery dates recorded and were therefore deemed ‘non-pCR’ per protocol for both efficacy and biomarker analyses.

**Table 1:** Baseline patient characteristics.

Characteristic	Ganetespib (*n* = 93)	Control (*n* = 140)
Median age (range), yr	48 (26–73)	48 (24–77)
Ethnicity, *n* (%)		
White	74 (80%)	109 (78%)
African American	13 (14%)	22 (16%)
Asian	6 (6%)	7 (5%)
Other/Mixed	0 (0%)	2 (1%)
HR Status, *n* (%)		
Positive	48 (52%)	72 (51%)
Negative	45 (48%)	68 (49%)
MammaPrint Status, *n* (%)		
MP.Hi1 (MP1)	32 (34%)	76 (54%)
MP.Hi2 (MP2)	61 (66%)	64 (46%)
Median Tumor Size by MRI (range), cm	3.8 (1.5–14)	3.9 (1.2–15)
Pretreatment SBR Grade		
I	1 (1%)	2 (1%)
II	10 (11%)	30 (21%)
III	46 (49%)	63 (45%)
N/A	36 (39%)	45 (32%)
Baseline node status, *n* (%)		
Palpable	26 (28%)	65 (46%)
Non-palpable	56 (60%)	64 (46%)
N/A	11 (12%)	11 (8%)

### Efficacy

**Fig. 2:**
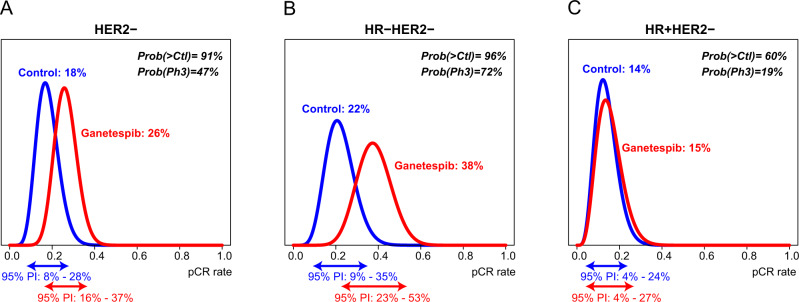
Estimated rate pf pCR with ganetespib versus the concurrent HER2-negative control. Final pCR probability distributions in ganetespib (red) and control (blue) arms, for **A** All HER2-negative participants, **B** HR-negative/HER2-negative participants, and **C** HR-positive/HER2-negative participants.

Ganetespib was evaluated in 3 predefined signatures: HER2-negative, HR-positive/HER2-negative, and triple negative (HR-negative/HER2-negative). Ganetespib did not meet the criteria for graduation in any of the three signatures prior to reaching maximum accrual to the arm. In all HER2-negative patients, estimates of pCR rates in the ganetespib (*n* = 93) versus control arms (*n* = 140) were 26% vs. 18% (95% Probability Interval (PI) of 16–37% vs. 8–28%) (Fig. 2, Supplementary Table 1). In triple negative disease, the estimated pCR rate was 38% (95% PI 23–53%) for those receiving ganetespib (*n* = 45) vs 22% (95% PI 9–35%) for the control arm (*n* = 68). In the HR-positive/HER2-negative breast cancer group, the estimated pCR rate was 15% (95% PI 4–27%) for ganetespib (*n* = 48) compared to 14% (95% PI 4–24%) for control (*n* = 72). Although the probability of ganetespib’s success in a 300-patient phase III neoadjuvant study was high in some signatures—72% in triple negative, 19% in HR-positive/HER2-negative and 47% in all HER2-negative—it fell short of the >85% threshold for graduation in each signature.

**Fig. 3:**
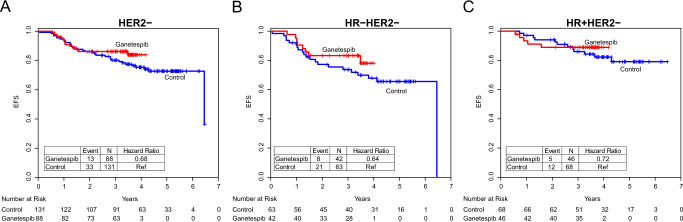
Event-free survival. Kaplan–Meier plots of event-free survival comparing ganetespib and control arms in **A** all HER2-negative participants, **B** triple negative participants, and **C** HR-positive/HER2-negative participants. Median follow-up in the overall (HER2-negative) population was 3.6 years.

We also performed an exploratory analysis of the relationship between treatment intervention and event-free survival (EFS). In the overall HER2-negative population, 88 patients in the ganetespib arm and 131 in the control arm had follow-up data, with median time of 3.6 years (Fig. 3). There were 13 EFS events observed in the ganetespib arm, and 33 in the control arm, yielding a hazard ratio of 0.68. Similarly, apparent benefit was seen in the HR-positive/HER2-negative and HR-negative/HER2-negative subtypes (hazard ratios 0.40 and 0.72, respectively). In addition, the relationship between pCR and event-free survival (EFS) was also evaluated (Supplementary Fig. 1). Of the 88 patients in the ganetespib arm, there was 1 EFS event of the 26 patients who achieved pCR, while there were 12 EFS events in the 62 patients without PCR, giving a hazard ratio of 0.19. Of the 131 patients in the control arm, no EFS events were observed in the 25 patients who achieved a pCR while there were 33 EFS events in those 106 non-pCR patients (hazard ratio 0). In both ganetespib and control arms, achieving pCR was highly associated with 3-year EFS, although this must be considered exploratory since the numbers of patients analyzed in both arms was small.

### Toxicity

The most common adverse events (AE) observed in either arm are shown in Table 2, which lists Grade 3/4 events occurring in ≥2% of an arm’s participants, as well as Grade 1/2 events that were observed in ≥20% of an arm’s participants. The most common AEs observed among the patients enrolled in the ganetespib group were fatigue (86%), nausea (79%), diarrhea (79%), alopecia (72%), peripheral sensorial neuropathy (72%) and constipation (57%); most events were grade 1 or 2. Except for diarrhea (81% vs. 42%, respectively) and neutropenia (28% vs. 16%, respectively), which had a higher incidence in the ganetespib arm, the occurrence of other AEs were similar between arms. The most common grade 3 or higher AEs among patients in the ganetespib group were neutropenia, leukopenia, diarrhea, peripheral sensorial neuropathy, anemia, ALT elevation and fever neutropenia. As expected, the addition of ganetespib led to an increase in the incidence of neutropenia, diarrhea, sensorial peripheral neuropathy, and ALT elevations. There were no grade 5 events in the ganetespib group.

**Table 2:** Adverse events occurring in ganetespib and control arms; Grade 3/4 observed in ≥2% of participants or Grade 1/2 ≥ 20% of participants; dose reductions and early discontinuations (number, % of participants in arm/treatment phase).

	Ganetespib (*n* = 93)		Control (*n* = 140)	
Adverse event	Grade 1–2	Grade 3–4	Grade 1–2	Grade 3–4
Neutrophil count (decrease)	6 (6.5%)	20 (21.5%)	9 (6.4%)	14 (10.0%)
White blood cell count (decrease)	5 (5.4%)	9 (9.7%)	7 (5.0%)	7 (5.0%)
Diarrhea	67 (72.0%)	8 (8.6%)	56 (40.0%)	3 (2.1%)
Peripheral sensory neuropathy	59 (63.4%)	8 (8.6%)	90 (64.3%)	2 (1.4%)
Anemia	22 (23.7%)	5 (5.4%)	19 (13.6%)	9 (6.4%)
Alanine aminotransferase (increase)	7 (7.5%)	5 (5.4%)	11 (7.9%)	3 (2.1%)
Febrile neutropenia	0 (0.0%)	3 (3.2%)	0 (0.0%)	12 (8.6%)
Stomatitis	33 (35.5%)	3 (3.2%)	43 (30.7%)	3 (2.1%)
Fatigue	77 (82.8%)	3 (3.2%)	122 (87.1%)	1 (0.7%)
Headache	43 (46.2%)	3 (3.2%)	61 (43.6%)	1 (0.7%)
Neutropenia	2 (2.2%)	2 (2.2%)	1 (0.7%)	2 (1.4%)
Vomiting	28 (30.1%)	2 (2.2%)	26 (18.6%)	0 (0.0%)
Arthralgia	33 (35.5%)	2 (2.2%)	35 (25.0%)	1 (0.7%)
Pulmonary embolism	0 (0.0%)	2 (2.2%)	0 (0.0%)	0 (0.0%)
Embolism	2 (2.2%)	2 (2.2%)	1 (0.7%)	0 (0.0%)
Nausea	72 (77.4%)	1 (1.1%)	106 (75.7%)	0 (0.0%)
Pain	19 (20.4%)	1 (1.1%)	18 (12.9%)	1 (0.7%)
Lymphocyte count (decrease)	3 (3.2%)	1 (1.1%)	3 (2.1%)	3 (2.1%)
Anorexia	30 (32.3%)	1 (1.1%)	30 (21.4%)	0 (0.0%)
Myalgia	25 (26.9%)	1 (1.1%)	36 (25.7%)	1 (0.7%)
Anxiety	10 (10.8%)	1 (1.1%)	36 (25.7%)	0 (0.0%)
Cough	22 (23.7%)	1 (1.1%)	35 (25.0%)	0 (0.0%)
Hypokalaemia	3 (3.2%)	0 (0.0%)	10 (7.1%)	4 (2.9%)
Bone pain	18 (19.4%)	0 (0.0%)	41 (29.3%)	3 (2.1%)
Pruritus	12 (12.9%)	0 (0.0%)	17 (12.1%)	1 (0.7%)
Alopecia	66 (71.0%)	0 (0.0%)	106 (75.7%)	0 (0.0%)
Constipation	53 (57.0%)	0 (0.0%)	74 (52.9%)	0 (0.0%)
Insomnia	35 (37.6%)	0 (0.0%)	55 (39.3%)	0 (0.0%)
Hot flush	32 (34.4%)	0 (0.0%)	57 (40.7%)	0 (0.0%)
Vision blurred	25 (26.9%)	0 (0.0%)	15 (10.7%)	0 (0.0%)
Dysgeusia	24 (25.8%)	0 (0.0%)	30 (21.4%)	0 (0.0%)
Dermatitis acneiform	24 (25.8%)	0 (0.0%)	28 (20.0%)	0 (0.0%)
Dyspnea	23 (24.7%)	0 (0.0%)	30 (21.4%)	0 (0.0%)
Nail discolouration	22 (23.7%)	0 (0.0%)	30 (21.4%)	0 (0.0%)
Rash maculo-papular	20 (21.5%)	0 (0.0%)	29 (20.7%)	0 (0.0%)
Gastrooesophageal reflux disease	18 (19.4%)	0 (0.0%)	30 (21.4%)	0 (0.0%)
Dose reductions, *n* (%)	16 (17.2%)		11 (7.9%)	
Early discontinuation, *n* (%)	37 (39.8%)		33 (23.6%)	
Toxicity	15 (16.2%)		10 (7.1%)	
Progression	12 (12.9%)		10 (7.1%)	
Other	10 (10.8%)		13 (9.3%)	
Time from treatment consent to surgery (days)				
Median (range)	165 (71–250)		165 (100–289)	
Follow-up time (years)				
Median (range)	3.4 (0.6–4.2)		4.1 (0.5–6.5)	

### Dose reductions and discontinuations

In the ganetespib arm, 16/93 (17.2%) required dose reductions compared to 11/140 (7.9%) in the control arm. Early discontinuations occurred in 37/93 (39.8%) in the ganetespib arm and 33/140 (23.6%) in the control arm. Table 2 provides additional details regarding reasons for early discontinuation by treatment arm. The median time from treatment consent to surgery was 165 days in both ganetespib and control arm.

### Assessment of biomarkers predictive of pCR

**Fig. 4:**
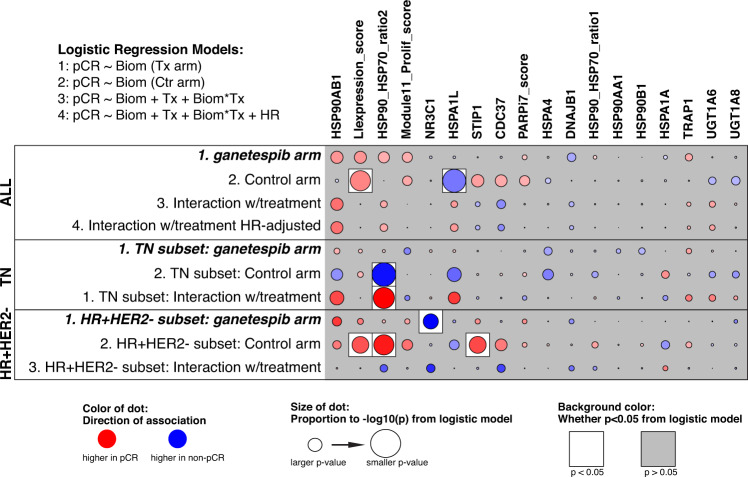
Analysis of pre-specified mechanism-of-action gene expression biomarkers for ganetespib response. Association dot-plot showing the level and direction of association between each gene (row) and pCR in the population/model as labeled (columns from right to left): G arm, control arm, interaction with treatment, interaction with treatment in a model adjusting for HR status; and analysis limited to the TN and HR+HER2- subsets. Key = red/blue dot indicates higher/lower levels ~ pCR; size of dot ~ strength of association (1/p likelihood ratio test), with dark outline/white background indicating *p* < 0.05 (likelihood ratio test) and gray background indicating *p* > 0.05 (likelihood ratio test). All biomarkers were assessed at the pre-treatment time point.

As shown in Fig. 4, in the overall study population, none of the 18 pre-specified biomarkers tested (including HSP90, GR/efflux, proliferation, DNA repair, and immune biomarkers) were significantly associated with pCR following ganetespib treatment (Likelihood ratio test *p* < 0.05, results shown in Supplementary Table 2). In receptor subset analysis, there were no significant associations with pCR in the TN group. In the HR-positive/HER2-negative subset, low levels of NR3C1 (glucocorticoid receptor) associated with pCR, but there were few responders in this group (*n* = 6).

## Discussion

We describe results from the I-SPY2 study arm examining HSP90 inhibition with ganetespib in combination with standard chemotherapy for high-risk HER2-negative stage 2–3 breast cancer. Preclinical data suggested efficacy of ganetespib in combination with taxanes with a mechanism of action based on inhibiting HSP90, an abundant chaperone protein involved in the folding of oncoproteins basally and during stress response^22^. Ganetespib did not meet criteria to graduate from I-SPY2 within the overall HER2-negative population, HR-positive/HER2-negative signature, or triple negative signature based on falling short of the required 85% threshold for predicted success in a well-powered clinical trial.

The primary goal of I-SPY2 is to identify novel agents worthy of further development in early-stage breast cancer in order to accelerate the pace of clinical development. While many agents have graduated in the past (neratinib, velaparib plus carboplatin, MK2206, pertuzumab, T-DM1 plus pertuzumab, and pembrolizumab), several agents have not met this goal (trebananib, ganitumab, and ganetespib completed accrual but did not graduate; pexidartinib and patritumab were halted).

Patients treated with ganetespib plus standard chemotherapy versus control achieved an estimated pCR rate of 26% versus 18%, which is generally lower than that observed in other arms of I-SPY2 involving HER2-negative patients. TNBC patients were the subgroup with the highest estimated pCR rates, with 38% for ganetespib vs. 22% for controls; however, the predicted probability of success of 72% was lower than the trial’s prespecified threshold of 85% probability threshold. Our results do not exclude the possibility that ganetespib could potentially show efficacy in TNBC in a larger trial or in combination with other investigational agents, such as targeted therapies or immunotherapy. HSP90 inhibitors have been studied for a few decades now and ganetespib is a potent second generation HSP90 inhibitor, so it is unlikely that next generations of HSP90 inhibitors would yield better results. HSP90 inhibition plus chemotherapy does not merit further study in breast cancer, but the use of HSP90 inhibitors plus anti-PD1 therapy and chemotherapy may warrant additional investigation.

Preclinical studies have shown great potential for HSP90 inhibition to enhance T-cell mediated anti-tumor responses via immunotherapy in melanoma^23^. Neoadjuvant anti-PD-1 therapy plus chemotherapy showed improved pCR and EFS compared to neoadjuvant chemotherapy alone in triple-negative breast cancer, however, FDA approval for neoadjuvant pembrolizumab occurred after the completion of the trial reported here. Further studies are needed to clarify if HSP90 inhibition can enhance immunotherapy in breast cancer^24^.

In I-SPY2, we have typically observed rates of pCR in the control group that are lower than those reported by other studies. Contributing to this is that, as per the I-SPY2 protocol, any patient who receives non-protocol therapy is considered non-pCR (for example, 5 patients in the triple negative patients on the control arm received carboplatin and therefore were assigned as non-pCR for the analyses). Another factor is I-SPY2’s use of the residual cancer burden (RCB) method for assessing pCR, which entails a more comprehensive evaluation of the resected surgical specimens and typically results in identifying cases of minimal residual disease.

For HR-positive/HER2-negative patients, the estimated pCR rate was 15% for ganetespib vs. 14% for control, corresponding to a 19% probability of success in a Phase III trial, far below the I-SPY2 criteria for graduation.

A recent Phase I trial of ganetespib, paclitaxel and trastuzumab in heavily pre-treated HER2-positive metastatic breast cancer (MBC) refractory to trastuzumab found synergistic clinical activity between HSP90 inhibition and HER2-targeted therapy combined with paclitaxel^25^. A Phase II trial of single agent ganetespib in heavily pre-treated MBC did not meet the prespecified objective response rate, however, some activity was observed in trastuzumab-refractory HER2-positive and in TNBC^13^. A retrospective analysis of potential biomarkers across multiple trials of HSP90 inhibition in metastatic breast cancer found that HER2 was the only immunohistochemistry biomarker predicting sensitivity to HSP90 inhibition^26^.

In advanced non-small cell lung cancer, ganetspib was evaluated in the phase 2 GALAXY-1 trial Ganetespib did not meet its primary endpoint of progression-free survival in either of two pre-specified subgroups: patients with elevated lactate dehydrogenase (eLDH) and mutated KRAS (mKRAS)^27^. However, based on an observed benefit in overall and progression-free survival in patients >6 months from the diagnosis of advanced disease, ganetespib went on to be evaluated in the phase 3 GALAXY-2 trial, but was terminated for futility. The addition of ganetespib to docetaxel did not improve survival for patients with advanced stage lung cancer with EGFR and ALK wild type status^14^.

These findings highlight the importance of identifying additional biomarkers predictive of response to ganetespib, and motivated the design of our biomarker studies in this trial. In addition to genes in the HSP90 pathway, we included three metabolic genes previously implicated in response to HSP90-inhibition, 2 signatures related to replicative stress and an immune signature. While the small sample size in I-SPY2 precludes definitive conclusions, none of these biomarkers or signatures was significantly associated with response to ganetespib, suggesting that further exploration is needed.

Recent results from animal models may explain the molecular mechanisms of resistance to ganetespib, such as HSP90 inhibition leading to upregulation of other heat shock proteins as well as receptor tyrosine kinases^28^. These other chaperone proteins are capable of interacting with and stabilizing known oncogenic drivers in breast cancer. AKT remains phospho-activated in ganetespib resistant tumors, suggesting the possibility of co-targeting AKT and HSP90 as a potentially better therapeutic strategy in future clinical trials^28^. Another concern is that HSP90 client levels return to normal within days of treatment and may stimulate heat-shock factor 1, a potent transcriptional regulator of carcinogenesis^29^. This plasticity may explain why our biomarker analysis of pre-treatment expression of genes associated with the HSP90 axis did not predict pCR, as pre-treatment gene expression cannot adequately capture HSP90 signaling dynamics. Analysis of biomarker and expression profiles from tumor biopsies obtained on ganetespib treatment may provide insights regarding response and resistance mechanisms.

Additionally, multiple HSP90 isoforms exist (including some induced by stress) that may also require targeting. Increased expression of HSP90 mRNA was found to be associated with poor prognosis in HER2-negative breast cancers, rendering them more aggressive^30^. Multiple lines of evidence reveal a program of plasticity among chaperone proteins with complex interactions with oncogenic drivers in breast cancer.

On the basis of these results in I-SPY2, further development of ganetespib seems unlikely to be successful as a neoadjuvant treatment for HR-negative early breast cancer. HSP90 inhibitors continue to be evaluated in clinical trials, including in HER2-positive metastatic breast cancer.

## Methods

### Study design

I-SPY2 is an ongoing, multicenter, open-label, adaptively randomized phase II multicenter trial of neoadjuvant therapy for locally advanced, early-stage breast cancer at high risk of recurrence (clinicaltrials.gov identifier NCT01042379)^31^. It is a platform trial evaluating multiple investigational arms in parallel, each evaluating an investigational agent/combination added to a backbone of standard of care neoadjuvant chemotherapy, which also serves as a common control arm.

The primary endpoint is pathological complete response (pCR), defined as the absence of invasive disease in breast and regional nodes (ypT0/is and ypN0) at time of surgery. The primary analysis is modified intent-to-treat, where all participants receiving allocated therapy are considered evaluable; those switching to non-protocol assigned therapy, forgoing surgery or withdrawing from the trial are assigned “non-pCR” status for analysis. Secondary endpoints include residual cancer burden (RCB), 3-year event-free survival (EFS) and distant relapse-free survival (DRFS). All patients are followed for long-term outcome and safety.

Baseline assessments of hormone receptor (HR), HER2 and MammaPrint^21^ (Agendia, Inc, Irvine, CA) status (Hi1 or Hi2, see Supplementary Fig. 2) are used to classify patients into one of 8 subtypes. Adaptive randomization in I-SPY2 preferentially assigns patients to experimental agents according to continuously updated Bayesian probabilities of rates of pCR for each subtype; 20% of patients are randomly assigned to control.

Arms ‘graduate’ from I-SPY2 when, in any of 10 clinically relevant signatures (based on HR, HER2 and MammaPrint), they reach the predefined efficacy threshold of 85% probability of success in a hypothetical, subtype-specific 300-patient, 1:1 confirmatory phase 3 trial. Agents are dropped for futility if the predicted probability of success in phase 3 is <10% in all signatures, or if enrollment in the arm reaches a predefined maximum. Additional details on the study design have been published previously^32,33^.

### Eligibility

Patients eligible for I-SPY2 are women ≥18 years, with stage II or III breast cancer and primary tumors >2.5 cm by clinical exam or >2.0 cm by imaging, with Eastern Cooperative Oncology Group performance status of 0 or 1^34^. MammaPrint low-risk HR-positive, HER2-negative patients are excluded from I-SPY2 as their lower risk of recurrence does not justify escalation of therapy^17^. All patients provide written informed consent prior to screening and again after randomization. Only HER2-negative patients were eligible for randomization to the ganetespib arm.

### Treatment

All participants in the ganetespib treatment or control arms received standard neoadjuvant chemotherapy consisting of 80 mg/m^2^ intravenous paclitaxel weekly for 12 weeks, followed by four cycles of intravenous 60 mg/m^2^ doxorubicin plus 600 mg/m^2^ cyclophosphamide (AC) every 2–3 weeks. Concomitant with paclitaxel, participants in the experimental arm also received infusions of 150 mg/m^2^ ganetespib in weeks 1, 2, 3, 5, 6, 7, 9, 10, and 11. Participants assigned to ganetespib received premedication with 10 mg intravenous dexamethasone and 25–50 mg diphenhydramine HCl (or therapeutic equivalents) and oral loperamide (2 mg hourly for 12 h beginning 1–2 h prior to ganetespib administration).

Definitive surgery followed AC, with lumpectomy or mastectomy at the discretion of the treating surgeon. Sentinel node dissection was allowed in node-negative patients, with axillary node dissection in node-positive patients according to NCCN and local practice guidelines^35^. Adjuvant treatment was not mandated by the trial, but was at the discretion of the treating oncologist. However, standard-of-care adjuvant therapy per NCCN guidelines was recommended.

Investigators are not blinded to randomization results, but are blinded to efficacy data until announcement that experimental regimens have exited the trial.

### Assessments

Core biopsies and breast MRIs were obtained per trial protocol and included four MRIs at baseline, after 3-weeks of paclitaxel-based treatment, between paclitaxel and AC and again following AC^32,33^. Dynamic contrast-enhanced (DCE) MRI was performed on a 3.0 T or 1.5 T MRI scanner using a dedicated breast coil and bilateral 3-dimensional, T1-weighted sequence with fat-suppression. Functional tumor volume (FTV) was measured from MR images by summing all pixels meeting defined thresholds for signal enhancement following gadolinium contrast injection^36^.

Surgical specimens were assessed for response by local pathologists trained in the residual cancer burden (RCB) method^37^. Biomarkers assessed include the 70-gene MammaPrint (MP) and TargetPrint HER2 gene expression assays using the 44 K full-genome microarray (Agendia)^21,38^. The MP poor prognosis designation was further stratified into high risk (MP1) and ultra-high risk (MP2) based on thresholds extrapolated from I-SPY1 patients who would have been eligible for I-SPY2^33^.

### Trial oversight

The trial was designed by the I-SPY2 study investigators. Madrigal Pharmaceuticals (Fort Washington, PA, formerly Synta Pharmaceuticals Corp.) provided funds and study drug but played no role in the study design, collection/analysis of data or manuscript preparation. The I-SPY2 Data and Safety Monitoring Board met monthly to review patient safety and study progress. The authors of the manuscript vouch for the accuracy and completeness of the data reported. The study complies with all local and national regulations regarding the use of human study participants and was conducted in accordance to the criteria set by the Declaration of Helsinki. The study received institutional review board approval at all clinical sites: University of California San Francisco Human Research Protection Program Institutional Review Board, The University of Alabama at Birmingham Office of the Institutional Review Board for Human Use, University of Minnesota Human Research Protection Program, University of California San Diego Human Research Protections Program Institutional Review Boards, University of Texas MD Anderson Cancer Center Clinical Institutional Review Board, Loyola University Chicago Health Sciences Division Institutional Review Board for the Protection of Human Subjects, Fred Hutchinson Cancer Research Center Institutional Review Board, UT Southwestern IRB, University of Southern California Health Sciences Institutional Review Board, University of Colorado Multiple Institutional Review Board, Mayo Clinic Institutional Review Boards, MedStar Health Research Institute-Georgetown University Oncology Institutional Review Board, University of Pennsylvania Office of Regulatory Affairs Institutional Review Board, and IRBs at Moffitt Cancer Center, University of Chicago, University of Kansas, Inova Health System.

### Statistical analysis

Probability distributions of pCR rates are continuously updated during the study, using a covariate-adjusted Bayesian longitudinal model based upon change in tumor volume by MRI (for those still undergoing treatment) and pathological response (for those who have completed surgery) with HR, HER2 and MammaPrint statuses as covariates. The model adjusts for time trends to allow comparisons against all enrolled I-SPY2 controls prior to the date randomization was stopped for the investigational arm. From these distributions, the probability that the pCR rate of the investigational arm is greater than control is assessed for each of the 10 clinically relevant biomarker signatures; and similarly, for the predictive probabilities of success in a future trial.

I-SPY2 uses contemporary controls adjusted for time trends. The initial statistical analyses in I-SPY 2 compared investigational arms with concurrently randomized controls. The approach applied to the first five investigational arms: neratinib, veliparib+carboplatin, trebananib, ganitumab, and Akt inhibitor MK2206. In September 2013 the FDA granted accelerated approval for pertuzumab+trastuzumab+docetaxel as neoadjuvant therapy for high risk HER2 + breast cancer. Our investigators and DSMB required dropping the I-SPY 2 control arm for HER2 + subtypes because it did not contain pertuzumab, which we did by amendment in early 2014. At the time pertuzumab+trastuzumab+paclitaxel (for the first 12 weeks of neoadjuvant therapy) was an investigational arm in the trial, but it had accrued only 6 patients with none through surgery.

We wanted to be able to use the results for the original control arm but were concerned about the possibility of a drift in the prognosis of patient population over time and within patient subtype. We built a model that we call “the time machine” that adjusts for the results over time within each arm, including results for the investigational arms as well as those for control. Having multiple arms in the trial with different time periods during which they are accruing patients enabled bridging across the different eras of trial accrual. The time machine discounts results from the past, with more discounting if they are further in the past. The mathematical basis and motivation was a statistical model for bridging eras in sports^39^. The model description follows.

The control rate for an investigational arm is adjusted to the time period when the arm was being randomized to patients. Each investigational arm is compared directly against its concurrently randomized controls. The time machine strengthens this comparison by bridging to earlier controls via a series of direct comparisons. These direct comparisons are the various comparisons of arms that have been randomized in the trial, including comparisons of investigational arms against each other as well as against controls. The strength of this borrowing depends on the time-period overlaps among the various arms, both control and investigational arms. The greater uncertainty associated with results during periods of relatively low accrual and when fewer arms are being randomized is incorporated into the final analyses of the various arms.

We explicitly incorporate terms in the model to account for potential time trends in the pCR rate; we account for molecular subtype and treatment as well. This is accomplished using time-dependent offset terms in a logistic model. Time is set to 0 at each analysis. We partition time in the past into bins of 90 days each. The index of the most recent bin, that for the previous 0–90 days, is 1. The index of the bin 91–180 days in the past is 2. And so on. Let *t*_*i*_ be the index of the bin for the randomization time of patient *i*.

We model time-trend parameters *δ*(*t*) within each bin *t*. These are additive parameters in the model for the log-odds ratio of pCR rate for each investigational arm compared with control. We use two sets of time-trend parameters, *δ*_+_(*t*) for HER2-positive and *δ*_–_(*t*) for HER2-negative. Consider patient *i* who has subtype (HR–, HER2 + , MP–) and was randomized 750 days before present. Her bin *t*_*i*_ is 9 and her time-trend offset is *δ*_+_(*9*).

Suppressing subscripts + and – for both HER2 + and HER2–, we set *δ* (*t*) = 0 for *t* = 1, 2, 3, 4. That means the previous year’s results count fully in the analysis. Further in the past, that is, for *t* > 4, {*δ*(*t*)} is a second-order normal dynamic linear model (NDLM)^40^. The NDLM uses the data within bins to estimate the respective log-odds ratios, but it also serves to smooth the effect across bins.

The time machine has the following structure for both HER2 + and HER2–, again suppressing the + and – subscripts:

$$\delta \left( 1 \right) = \delta \left( 2 \right) = \ldots = \delta \left( 4 \right) = 0$$

$$\delta \left( 5 \right)\sim N\pi \left( {\mu _0,\tau _0^2} \right)$$

$$\delta \left( 6 \right) - \delta (5)\sim N\left( {\mu _1,\tau _1^2} \right)$$

$$\delta (t) - 2\delta (t - 1) + \delta (t - 2)\sim N(0,\tau ^2)\,{{{\mathrm{for}}}}\,{\it{t}}\, > \,6$$

$$\tau ^2\sim IG(\alpha ,\beta )$$

In this notation, *N*(*μ*, *σ*^*2*^) refers to a normal distribution with mean *μ* and standard deviation *σ* and *IG* stands for inverse gamma. The parameters of the prior distributions are *μ*_*0*_ = *μ*_*1*_ = 0, $\tau _0^2 = \tau _1^2 = 0.001$, *α* = 1, *and β* = 0.001.

Kaplan-Meier survival curves for each arm were generated, with hazard ratios by Cox proportional hazard modeling. Statistics regarding this exploratory EFS analysis, assessed in March 2019, are descriptive only, as sample sizes are small and I-SPY2 is not powered for EFS or other survival endpoints.

### Biomarker analysis

Biomarker analyses were performed using Agendia 44 K full-genome microarrays assayed on biopsies at the pre-treatment time point for ganetespib and control population. We evaluated 10 genes involved in HSP90 pathway signaling (HSP90A-A1,AB1,B1; TRAP1; DNAJB1; HSPA1-A,L; HSPA4; STIP1, CD37), the HSP90/HSP70 ratio, 3 metabolism-related genes (glucocorticoid receptor and efflux genes; NR3C1; UGT1A6,8), two signatures representing different forms of replicative stress (DNA repair deficiency PARPi7_score^41^) and proliferation (Module11_prolif_score^42^), and 1 immune signature (LIexpression_score^43^) as biomarkers of ganetespib response. These pre-specified biomarkers were selected based on published evidence of association to HSP90 pathway signaling or/and response to HSP90-inhibition in at least one cancer type/model^15–20^. In pre-specified analyses, we used logistic regression to assess biomarker association with pCR. A biomarker was considered a specific predictor of ganetespib response if it associated with response in the ganetespib arm but not the control arm, and if the biomarker-by-treatment interaction term was significant (likelihood ratio test, *p* < 0.05). These analyses were also performed adjusting for HR status as a covariate, and within receptor subsets, sample size permitting. All computation was performed in the R programming environment (version 3.3.3).

## Supplementary information

Supplementary Information

## Data Availability

Clinical datasets that support the figures presented in this manuscript are available upon request by email to ispyadmin@ucsf.edu. Biomarker data will be uploaded to a public database and made available prior to publication.

## Code availability

Biomarker data analysis was performed using R version 3.3.3 and Bioconductor; no custom code was generated. The randomization engine and Bayesian analytic software used in efficacy analysis are used under license from Berry Consultants, L.L.C.; requests for code should be directed to don@berryconsultants.com.
