# Supplementary material for: Safety and efficacy of HSP90 inhibitor ganetespib for neoadjuvant treatment of stage II/III breast cancer
Source: NPJ Breast Cancer. 2022 Dec 1;8:128. doi: 10.1038/s41523-022-00493-z (PMC9715670; doi:10.1038/s41523-022-00493-z)

Supplementary Information

Safety and efficacy of neoadjuvant HSP90 inhibitor ganetespib in stage II/III breast cancer in the adaptively randomized I-SPY2 trial

Lang JE, et al.

Supplementary Table 1: Final predictive probabilities of success of ganetespib with paclitaxel followed by anthracyclines in HER2- signatures.

	Estimated Rate of pCR % (95% Probability Interval)			
**Biomarker Signature**	**ganetespib** n=93	**control** n=140	**Prob. Superior to Control, %**	**Predictive Prob. of Success in Phase III Trial, %**
HER2-	26 (16 - 37)	18 (8 – 28)	91	47
HR- HER2-	38 (23 - 53)	22 (9 - 35)	96	72
HR+ HER2-	15 (4 - 27)	14 (4 - 24)	60	19

Supplementary Table 2: Association of prespecified biomarkers with pCR in overall population and HR-negative/HR-negative (TN, triple negative) and HR-positive/HER2-negative subtypes.

	Overall Population					TN subset					HR+HER2-				
	**Ganetespib arm**		**Control arm**			**Ganetespib arm**		**Control arm**			**Ganetespib arm**		**Control arm**		
**Biomarker**	OR/unit inc	LR p	OR/unit inc	LR p	Biomarker x treatment int: LR p	OR/unit inc	LR p	OR/unit inc	LR p	Biomarker x treatment int: LR p	OR/unit inc	LR p	OR/unit inc	LR p	Biomarker x treatment int: LR p
**HSP90; single gene**															
HSP90AA1	1.02	0.934	0.932	0.752	0.78	0.728	0.376	0.958	0.891	0.569	1.02	0.953	0.904	0.758	0.81
HSP90AB1	1.59	0.074	0.861	0.5	0.0719	1.37	0.285	0.623	0.0848	0.0517	2.38	0.14	1.79	0.168	0.707
HSP90B1	0.985	0.942	1.11	0.67	0.71	0.718	0.25	0.819	0.616	0.788	1.03	0.939	1.24	0.536	0.726
TRAP1	1.46	0.211	1.07	0.748	0.401	1.49	0.314	0.848	0.562	0.248	1.1	0.861	1.42	0.279	0.692
DNAJB1	0.686	0.158	0.955	0.827	0.337	0.83	0.596	0.871	0.614	0.914	0.621	0.347	1.14	0.692	0.318
HSPA1A	0.842	0.434	0.897	0.634	0.841	1.03	0.909	1.62	0.193	0.34	1.08	0.873	0.627	0.165	0.342
HSPA1L	0.854	0.58	0.554	**0.00772**	0.242	1.23	0.555	0.519	0.0503	0.0791	0.697	0.533	0.618	0.123	0.858
HSPA4	0.901	0.665	0.796	0.306	0.707	0.672	0.175	0.582	0.101	0.755	1.11	0.834	1.19	0.601	0.91
STIP1	1.08	0.775	1.5	0.0713	0.356	0.881	0.725	1.14	0.651	0.576	1.63	0.332	2.22	**0.0276**	0.627
CDC37	0.869	0.63	1.46	0.0794	0.152	0.975	0.952	1.18	0.578	0.712	0.793	0.654	1.79	0.08	0.176
**GR/efflux; single gene**															
UGT1A6	1.07	0.762	0.72	0.203	0.27	1.13	0.65	0.657	0.251	0.24	0.879	0.825	0.823	0.587	0.927
UGT1A8	0.879	0.642	0.72	0.161	0.601	0.942	0.839	0.633	0.225	0.417	0.605	0.451	0.84	0.573	0.664
NR3C1	0.867	0.572	0.929	0.742	0.837	1.2	0.563	0.98	0.943	0.634	0.325	0.04	0.828	0.594	0.163
**HSP90 ratio**															
HSP90_HSP70_ratio1	1.18	0.456	1.06	0.798	0.745	0.866	0.646	0.677	0.257	0.603	0.953	0.912	1.47	0.258	0.423
HSP90_HSP70_ratio2	1.42	0.0923	0.979	0.927	0.232	1.18	0.537	0.349	**0.00686**	**0.0116**	1.31	0.496	2.71	**0.0142**	0.2
**Immune signature**															
LIexpression_score	1.58	0.0761	1.7	**0.0141**	0.84	1.27	0.435	1.36	0.292	0.883	1.78	0.278	2.12	**0.0284**	0.785
**Replicative stress (proliferation) signature**															
Module11_Prolif_score	1.43	0.115	1.45	0.131	0.976	0.586	0.237	1.02	0.947	0.331	1.47	0.33	1.84	0.104	0.694
PARPi7_score	1.26	0.351	1.42	0.112	0.719	0.87	0.654	1.28	0.43	0.381	1.61	0.364	1.42	0.361	0.844

Supplementary Figure 1: Kaplan Meier lots of event-free survival in ganetespib and control arms, displayed by post-surgical pCR status in: (A) all HER2-negative participants; (B) triple negative participants; (C) HR-positive/HER2-negative participants.

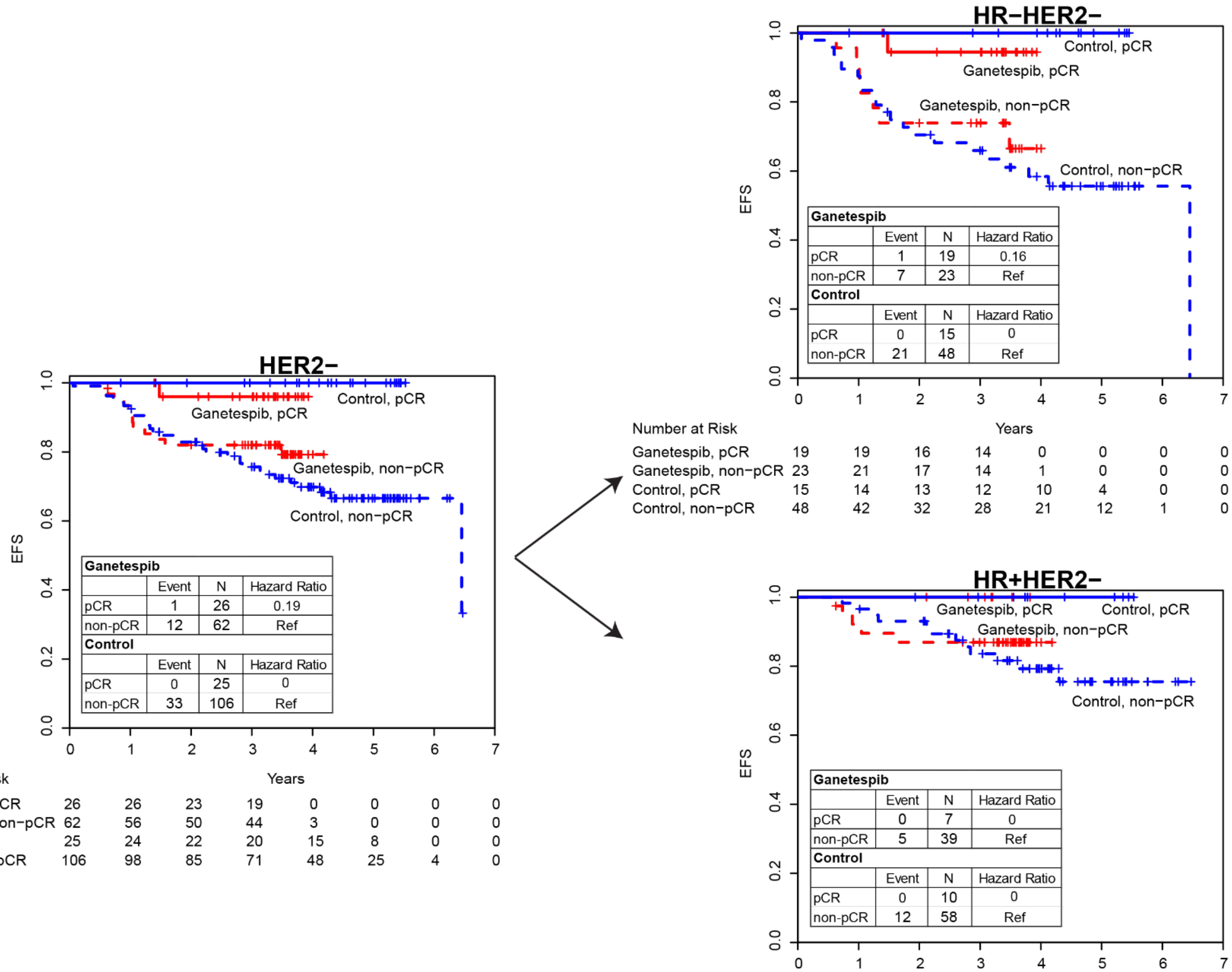

HER2−

Number at Risk	0	1	2	3	4	5	6	7
Ganetespib, pCR	26	26	23	19	0	0	0	0
Ganetespib, non−pCR	62	56	50	44	3	0	0	0
Control, pCR	25	24	22	20	15	8	0	0
Control, non−pCR	106	98	85	71	48	25	4	0

HR−HER2−

Number at Risk	0	1	2	3	4	5	6	7
Ganetespib, pCR	19	19	16	14	0	0	0	0
Ganetespib, non−pCR	23	21	17	14	1	0	0	0
Control, pCR	15	14	13	12	10	4	0	0
Control, non−pCR	48	42	32	28	21	12	1	0

HR+HER2−

Number at Risk	0	1	2	3	4	5	6	7
Ganetespib, pCR	7	7	7	5	0	0	0	0
Ganetespib, non−pCR	39	35	33	30	2	0	0	0
Control, pCR	10	10	9	8	5	4	0	0
Control, non−pCR	58	56	53	43	27	13	3	0

Supplementary Figure 2: Definition of MP1 and MP2 subtype. The figure depicts a heat map representation of the 70 genes in the MammaPrint score. The rows are the patients, and the columns are each of the 70 genes. The cut point of -0.154 for MP1 vs MP 2 is the midpoint of I-SPY 1 patient MammaPrint results that would have been eligible for I-SPY 2. The MP1 to MP2 threshold on the current commercially available MP test translates to –0.569, after an adjustment of the high versus low threshold to 0.00 (numerical subtraction of 0.415). The prevalence for each of the subtypes (as of August 2015) in the I–SPY 2 TRIAL is listed along the right hand border of the figure.

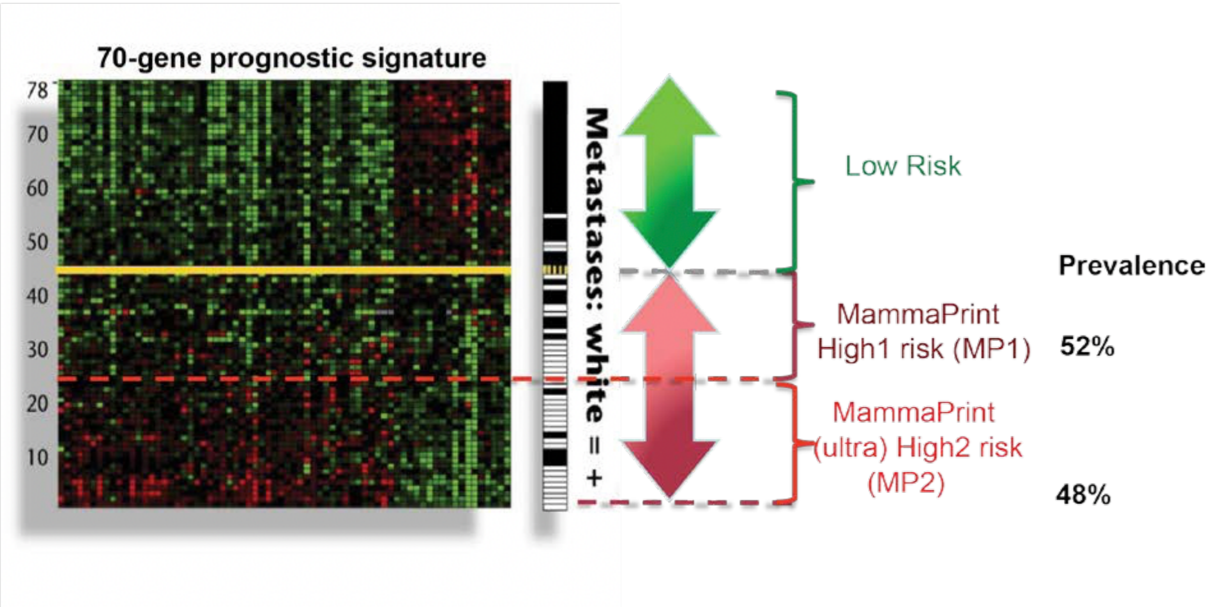

Supplement: Supplementary file 1 — Supplementary Information [file 41523_2022_493_MOESM1_ESM.pdf]
